# Comparison of one-year postoperative outcomes between PreserFlo MicroShunt and trabeculectomy in Japanese patients with open angle glaucoma: a retrospective cohort study using propensity score matching

**DOI:** 10.1007/s10384-026-01374-9

**Published:** 2026-05-30

**Authors:** Kana Katakami, Mari Sakamoto, Nanami Okuzumi, Yuto Iwaki, Sotaro Mori, Yuko Yamada-Nakanishi, Makoto Nakamura

**Affiliations:** Division of Ophthalmology, Department of Surgery, Kobe University Graduate School of Medicine, 7-5-2, Kusunoki-cho, Chuo-ku, Kobe, 650-0017 Japan

**Keywords:** PreserFlo MicroShunt, Trabeculectomy, Glaucoma

## Abstract

**Purpose:**

To compare one‑year postoperative outcomes between PreserFlo MicroShunt (PMS) and trabeculectomy (TLE) as primary filtration surgery in Japanese patients with open‑angle glaucoma using propensity score matching.

**Study design:**

Retrospective, single‑center observational cohort study.

**Methods:**

Patients with open‑angle glaucoma who underwent stand‑alone PMS implantation or TLE at Kobe University Hospital were included. After applying exclusion criteria, 102 eyes in each of 2 groups were matched 1:1 using propensity scores based on age, sex, lens status, glaucoma subtype, preoperative intraocular pressure (IOP), number of glaucoma medications, and best‑corrected visual acuity (BCVA). The primary outcome was IOP at 12 months postoperatively. Secondary outcomes included longitudinal IOP changes, medication use, BCVA, corneal endothelial cell density (ECD), surgical success rates, postoperative complications, and additional surgery. Mixed‑effects models and Kaplan–Meier analyses were performed.

**Results:**

At 12 months, mean IOP was significantly lower in the TLE group than in the PMS group (12.3 vs 14.1 mmHg, P = 0.042). Both groups showed significant IOP reduction from baseline. The proportion of medication‑free eyes did not differ between groups. BCVA worsened transiently after surgery and partially recovered but remained worse than baseline at 12 months, with no between‑group differences. ECD decreased modestly in both groups without significant differences. Surgical success rates were comparable across all criteria. Postoperative complications, particularly hypotony‑related events, were more frequent in the TLE group, and persistent hypotony was observed only after TLE.

**Conclusions:**

PMS demonstrated a more favorable safety profile with fewer hypotony‑related complications, whereas TLE achieved greater IOP reduction at 12 months. Surgical success rates were comparable. PMS may represent a less invasive option, while TLE may be preferable when maximal IOP lowering is required.

**Supplementary Information:**

The online version contains supplementary material available at https://doi.org/10.1007/s10384-026-01374-9.

## Introduction

Lowering intraocular pressure (IOP) remains the only proven treatment for glaucoma, and several surgical options are available to achieve this goal. Among these, trabeculectomy (TLE) is regarded as the gold standard because of its strong IOP-lowering efficacy. However, TLE is associated with postoperative complications such as hypotony and infection, as well as complex postoperative management. In recent years, minimally invasive and safe surgical techniques have been developed, and their use has been increasing [1, 2]. The PreserFlo MicroShunt (PMS; Santen) is a novel subconjunctival filtration device approved in Japan in 2022. Like TLE, PMS lowers IOP by diverting aqueous humor into the subconjunctival space to form a filtering bleb. However, PMS is less invasive because it does not require scleral or iris tissue resection [3]. Previous studies report that PMS offers shorter operative times [4, 5] requires fewer postoperative visits and less intensive postoperative management [5, 6], and may reduce the postoperative burden on patients. Furthermore, most studies associate PMS with fewer hypotony-related complications than TLE [4, 5, 7]. On the other hand, many studies show that PMS has a lower IOP-lowering effect compared to TLE [7–13].

Whether PMS should be selected as the initial filtration surgery based on its safety profile, or whether TLE should be preferred for its superior IOP-lowering effect, remains unclear. Although randomized controlled trials (RCT) comparing PMS and TLE have been conducted overseas [9, 11, 12], no such trials have been performed in Japanese patients. Given the established advantages of PMS in invasiveness and safety, conducting a new RCT in Japan may be impractical. Therefore, we conducted a retrospective study comparing one-year postoperative outcomes of PMS and TLE as primary filtration surgical procedures in Japanese patients with open-angle glaucoma, using propensity score matching to adjust for baseline differences.

## Methods

### Study design

This retrospective, single-center observational cohort study was conducted at Kobe University Hospital. The study protocol was approved by the Institutional Review Board of Kobe University Hospital (approval no. B250176) and adhered to the tenets of the Declaration of Helsinki. Study details were posted on the hospital website and on institutional notice boards, allowing patients the opportunity to opt out. Because the study used fully anonymized data, the requirement for written informed consent was waived. Patient privacy was strictly protected throughout the study period.

### Patients

We included patients with open‑angle glaucoma who underwent either stand-alone PMS implantation (April 2023–June 2024) or trabeculectomy (TLE) (April 2021–February 2023) as their initial filtration surgery at Kobe University Hospital. PMS was introduced at our institution in April 2023, after which PMS procedures increased and TLE procedures decreased. To minimize selection bias, TLE cases were included only from the period before PMS introduction. Exclusion criteria were: age <18 years, TLE combined with amniotic membrane transplantation, follow‑up period < 6 months after PMS/TLE, and childhood glaucoma. Whenever both eyes were eligible, the first operated eye was included.

### Data collection

The following variables were extracted from medical records: age, sex, glaucoma subtype, treatment history, number of glaucoma medications, lens status, best‑corrected visual acuity (BCVA), mean deviation (MD) on Humphrey visual field testing, IOP measured by Goldmann applanation tonometry, central corneal endothelial cell density (ECD), perioperative complications, and additional surgical interventions. Combination eye drops and oral acetazolamide were counted as two medications. IOP, number of medications, BCVA, and ECD were recorded at baseline and postoperative follow‑up visits.

### Propensity score matching

Logistic regression was performed with surgical procedure (PMS or TLE) as the dependent variable and age, sex, lens status, glaucoma subtype, preoperative IOP, number of preoperative medications, and preoperative BCVA as independent variables. MD was excluded because visual field test programs varied (24-2, 24-2C, 30-2, 10-2), and some patients lacked MD data due to central visual field defects. PMS and TLE cases were matched 1:1 using propensity scores with a caliper of 0.02 (0.15 SD of the propensity score), and covariate balance was assessed using standardized mean differences.

### Outcome measures

The primary outcome was IOP at 12 months postoperatively. Secondary outcomes included longitudinal changes in IOP, number of glaucoma medications, BCVA, and ECD; incidence and characteristics of surgical complications; and additional glaucoma surgeries.

Kaplan–Meier analysis was performed to evaluate the surgical success rate up to 12 months postoperatively. Three IOP criteria were defined as follows: (A) 6 mmHg ≤ IOP ≤ 18 mmHg, (B) 6 mmHg ≤ IOP ≤ 15 mmHg, and (C) 6 mmHg ≤ IOP ≤ 12 mmHg. For all criteria, an IOP reduction of at least 20% from baseline was required. Surgical failure was defined as IOP exceeding the defined criteria at two consecutive visits; IOP < 6 mmHg at two consecutive visits after 3 months postoperatively; the need for additional glaucoma surgery; or loss of light perception. For failure due to deviation from the IOP criteria, the date of failure was defined as the first of the two consecutive visits. Patients who were lost to follow-up after 6 months postoperatively were censored at their last visit. At our institution, early postoperative bleb scarring is managed with mitomycin C-assisted bleb revision rather than needling. This procedure, which has been described [14], involves advancing a blunt needle under the scleral flap through a small posterior conjunctival incision to restore aqueous humor outflow. In the present study, bleb revision performed at our institution was not considered an additional surgery, in the same manner as needling at other institutions.

In addition, based on the report by Baker et al. [9], an additional analysis was performed using the RCT criterion, defined as an IOP reduction of ≥20% from baseline at 12 months postoperatively, with surgical failure defined in the same manner as described above.

### Statistical analysis

Between‑group comparisons were performed using Fisher’s exact test or Wilcoxon signed-rank test as appropriate. Within‑group comparisons of pre‑ and postoperative values used the Wilcoxon signed‑rank test. Continuous outcomes (IOP, BCVA, and ECD) were analyzed using linear mixed‑effects models and are presented as estimated marginal means with 95% confidence intervals. To account for the dependency within propensity score–matched pairs and repeated measurements within individuals, we fitted a linear mixed-effects model with a random intercept for matched pairs (paired ID), while within-subject correlation was modeled using an AR(1) covariance structure (SUBJECT=ID). Surgical procedure, time, and their interaction were included as fixed effects.

The number of glaucoma medications exhibited a highly skewed distribution with a pronounced floor effect after surgery; therefore, medication counts were summarized using medians (IQR), and longitudinal medication outcomes were described as the proportion of medication‑free eyes with 95% confidence intervals (Wilson method). Statistical analyses were performed using MedCalc version 23.2.8 and SPSS version 31.0.1.0. A two‑sided P value <0.05 was considered statistically significant.

### Surgical procedures

PMS implantation was performed according to manufacturer instructions. After sub‑Tenon anesthesia with 2% lidocaine, a clear‑corneal stay suture (6‑0 Vicryl) was placed to rotate the eye downward, and a fornix‑based conjunctival incision was created superiorly. Following diathermy as needed, 0.04% MMC was applied for 3 minutes and irrigated. A paracentesis was created, and a double‑step knife was used to enter the anterior chamber 3 mm posterior to the limbus. The PMS tube was inserted into the scleral tunnel with the bevel facing the cornea until the fin was secured under the scleral pocket. After confirming aqueous outflow, Tenon’s capsule and conjunctiva were closed with 10‑0 nylon or 10‑0 Vicryl.

TLE was performed as previously described [15]. Briefly, a fornix‑based conjunctival incision was made, followed by creation of a 3.5–4 mm square half‑thickness scleral flap. After applying 0.04% MMC for 3 minutes and irrigating, a second scleral flap and trabecular tissue were excised. Peripheral iridectomy was performed, and the scleral flap was sutured with 5–7 10‑0 nylon sutures. Conjunctiva was closed with 10‑0 nylon.

Postoperative topical therapy for both procedures included betamethasone and antibiotic drops four to six times daily, tapered according to bleb appearance and IOP.

## Results

**Fig. 1:**
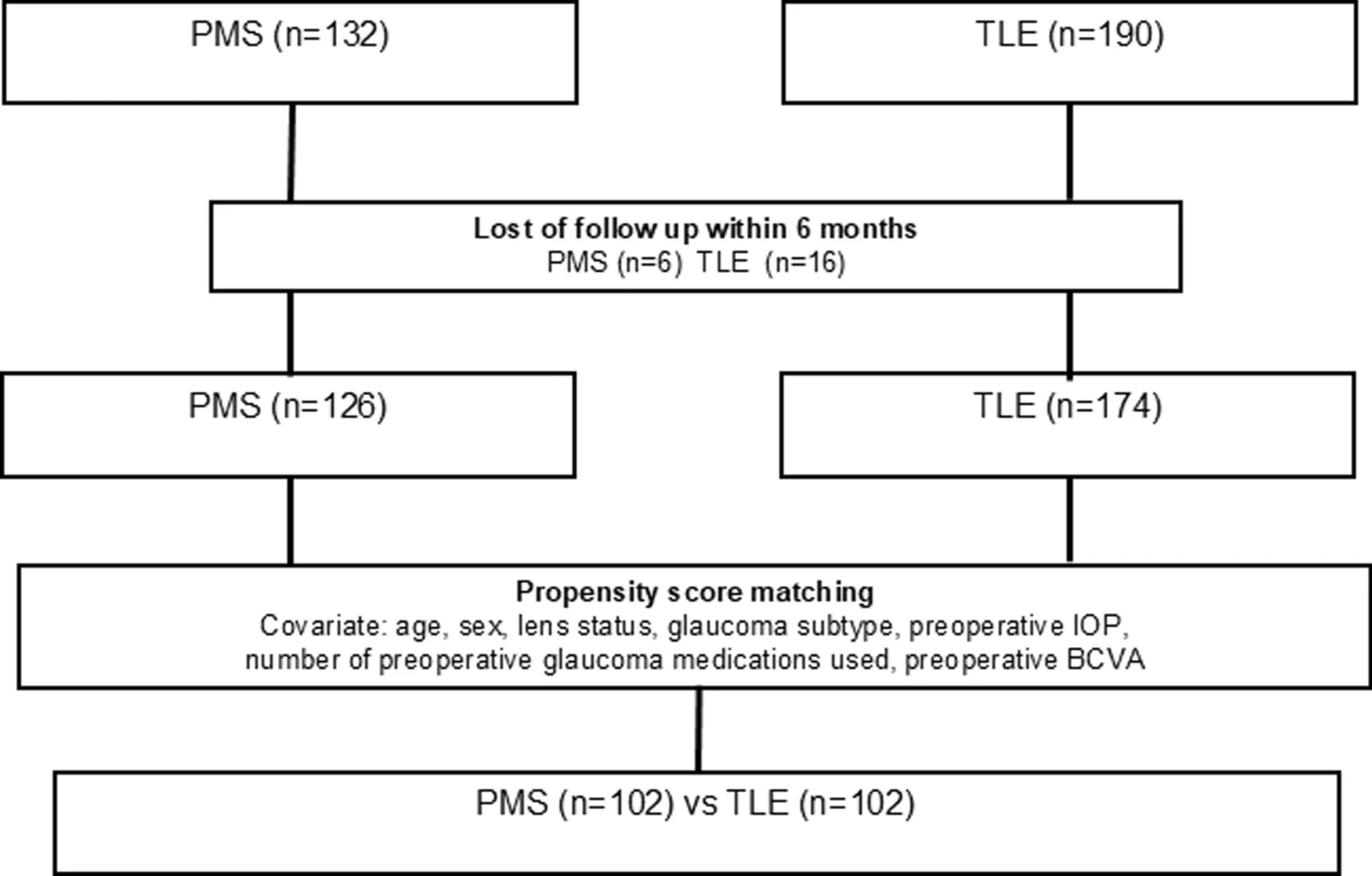
Composition of enrolled patients. PMS, Preserflo MicroShunt; TLE, trabeculectomy; IOP, intraocular pressure; BCVA, best corrected visual acuity.

A total of 132 eyes underwent PMS and 190 eyes underwent TLE during the study period. After excluding cases with <6 months of follow‑up, 126 PMS eyes and 174 TLE eyes were included (Fig. 1). Baseline characteristics before matching are shown in Online Resource 1. Significant differences were observed between groups in age, preoperative IOP, and number of preoperative medications.

After propensity score matching, 102 eyes were included in each group. Table 1 summarizes the matched baseline characteristics. No significant differences were observed for any variable, and standardized mean differences were acceptably low. In both groups, approximately 65% of eyes had primary open‑angle glaucoma (POAG) and approximately 25% had secondary glaucoma, including exfoliative glaucoma (XFG). MD values were not used in subsequent analyses because of variability in test programs and missing data in eyes with central visual field defects.

**Table 1:** Patients’ demographic and ophthalmic data after propensity score matching

	PMS (n=102)	TLE (n=102)	*P* value*	SMD
Age (years)	73.0 (61.0 to 80.0)	70.0 (63.0 to 78.0)	0.40	0.07
Sex (male, %)	57 (55.9%)	56 (54.9%)	0.89^†^	0.02
Glaucoma type (POAG, %)	68 (66.7%)	65 (63.7%)	0.77^†^	0.08
POAG	68	65	-	-
XFG	23	25	-	-
Secondary (excluding XFG)	11	12	-	-
preoperative IOP (mmHg)	22.0 (18.0 to 28.0)	22.5 (17.0 to 29.0)	0.76	0.08
preoperative MD (dB)	-15.4 (-22.7 to -9.8)	-16.6 (-21.4 to -11.3)	0.92	0.06
preoperative medication score	4.0 (3.0 to 5.0)	5.0 (4.0 to 5.0)	0.54	0.02
preoperative ECD (cells/mm^2^)	2287.5 (1903.0 to 2637.0)	2271.5 (1912.0 to 2552.0)	0.73	0.1
preoperative BCVA (logMAR)	0.023 (0.000 to 0.222)	0.046 (-0.079 to 0.301)	0.70	0.06
Lens status (phakia, %)	24 (23.5%)	21 (20.6%)	0.74^†^	0.13

Data are shown as median (interquartile range). * Wilcoxon rank sum test, ^†^ Fisher's exact test

PMS, PreserFlo MicroShunt; TLE, trabeculectomy; SMD, standardized mean difference; POAG, primary open angle glaucoma; XFG, exfoliative glaucoma; IOP, intraocular pressure; MD, mean deviation; ECD, corneal endothelial cell density; BCVA, best corrected visual acuity; log MAR, logarithm of the minimum angle of resolution

**Fig. 2:**
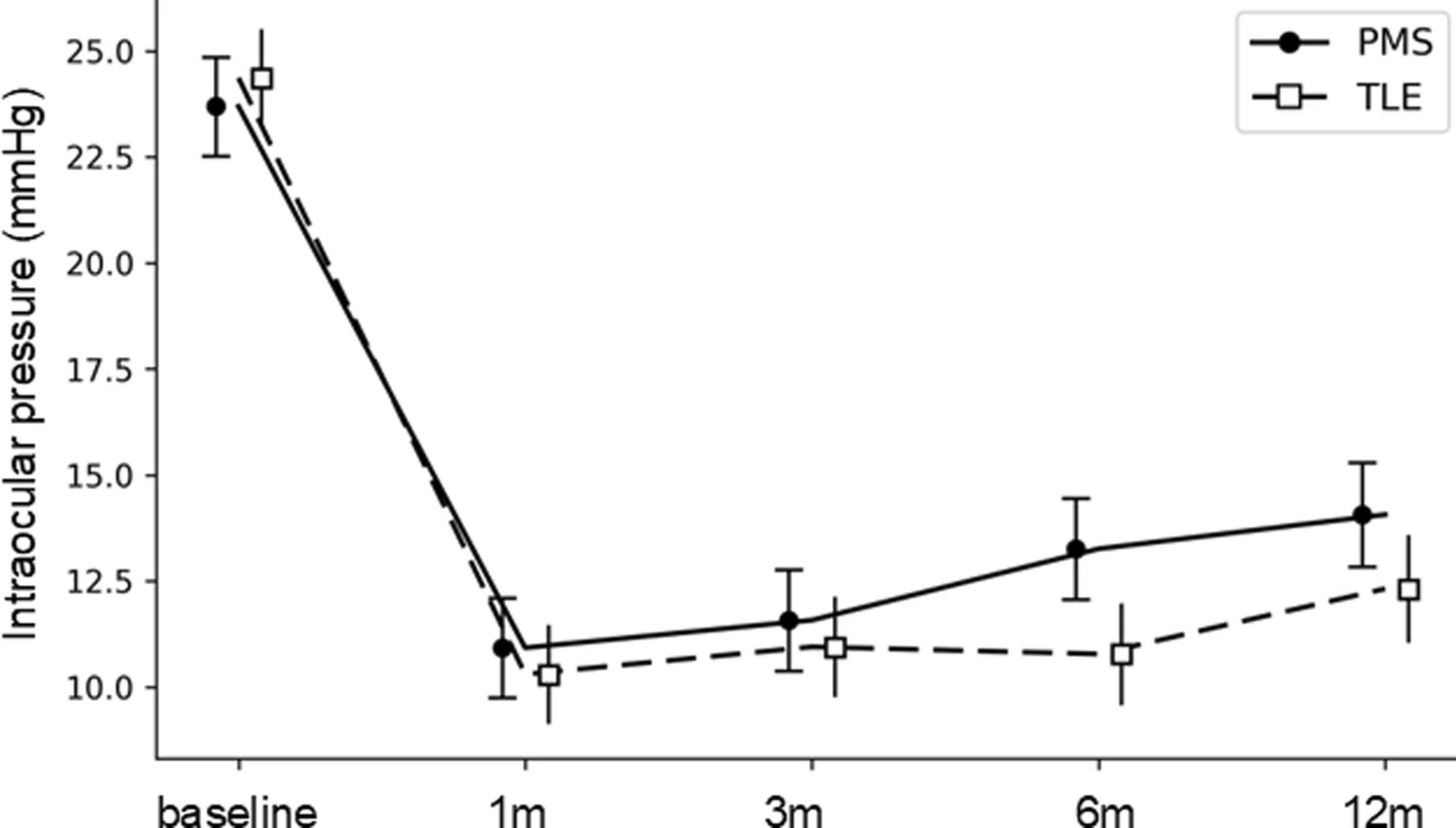
Estimated marginal mean (EMM) intraocular pressure over time. Lines show EMMs with 95% confidence intervals at preoperative, 1‑, 3‑, 6‑, and 12‑month postoperative visits. PMS is plotted as a solid line with filled circles and TLE as a dashed line with open squares; groups are slightly offset horizontally for clarity. Values are derived from a linear mixed‑effects model with fixed effects of surgery, time, and their interaction; an AR(1) within‑subject covariance structure; and Satterthwaite’s degrees‑of‑freedom approximation. PMS, PreserFlo Microshunt; TLE, trabeculectomy.

**Primary endpoint (IOP at 12 months).** At 12 months postoperatively, the estimated marginal mean IOP was 14.06 mmHg (95% CI, 12.83–15.30) in the PMS group and 12.32 mmHg (11.04–13.59) in the TLE group (Table 2). The between‑group difference (PMS−TLE) was 1.75 mmHg (95% CI, 0.06–3.44) and reached statistical significance after Bonferroni adjustment (*P* = 0.042), indicating lower IOP in the TLE group at 12 months (Fig. 2).

**Table 2:** Estimated marginal mean intraocular pressure and between‑group differences at each postoperative time point

Time	Group	EMM IOP (mmHg)	95%CI	Between group differences of EMM	95%CI	*P* value
baseline	PMS	23.69	22.52 to 24.85	-0.67	-2.23 to 0.88	0.397
	TLE	24.36	23.19 to 25.52			
Month1	PMS	10.92	9.75 to 12.09	0.63	-0.94 to 2.19	0.433
	TLE	10.30	9.13 to 11.47			
Month3	PMS	11.58	10.38 to 12.77	0.63	-0.97 to 2.22	0.439
	TLE	10.95	9.76 to 12.13			
Month6	PMS	13.26	12.07 to 14.45	2.48	0.88 to 4.08	**0.002**
	TLE	10.78	9.58 to 11.98			
Month12	PMS	14.06	12.83 to 15.30	1.75	0.06 to 3.44	**0.042**
	TLE	12.32	11.04 to 13.59			

EMM, estimated marginal mean; PMS, PreserFlo Microshunt; TLE, trabeculectomy. Data is expressed in mmHg

Between group difference is defined as PMS-TLE. Values are derived from a linear mixed-effects model with fixed effect of surgery, time, and their interaction; an AR(1) within subject covariance structure; and Satterthwaite’s degrees-of-freedom approximation. *P*-values are Bonferroni-adjusted for multiple comparisons across time-point contrasts

Bold values indicate statistically significant between‑group differences (Bonferroni‑adjusted P < 0.05)

**Time‑course of IOP.** In both groups, estimated postoperative IOPs were lower than baseline at all visits (1, 3, 6, and 12 months; Fig. 2). No significant between‑group differences were observed at 1or 3 months. At 6 months, IOP was significantly lower in the TLE group, with a between‑group difference of 2.48 mmHg (95% CI, 0.88–4.08; *P*=0.002). At 12 months, the difference remained in the same direction (+1.75 mmHg) and was significant after Bonferroni adjustment (95% CI, 0.06–3.44; *P*=0.042) (Table 2 and Fig. 2).

**Fig. 3:**
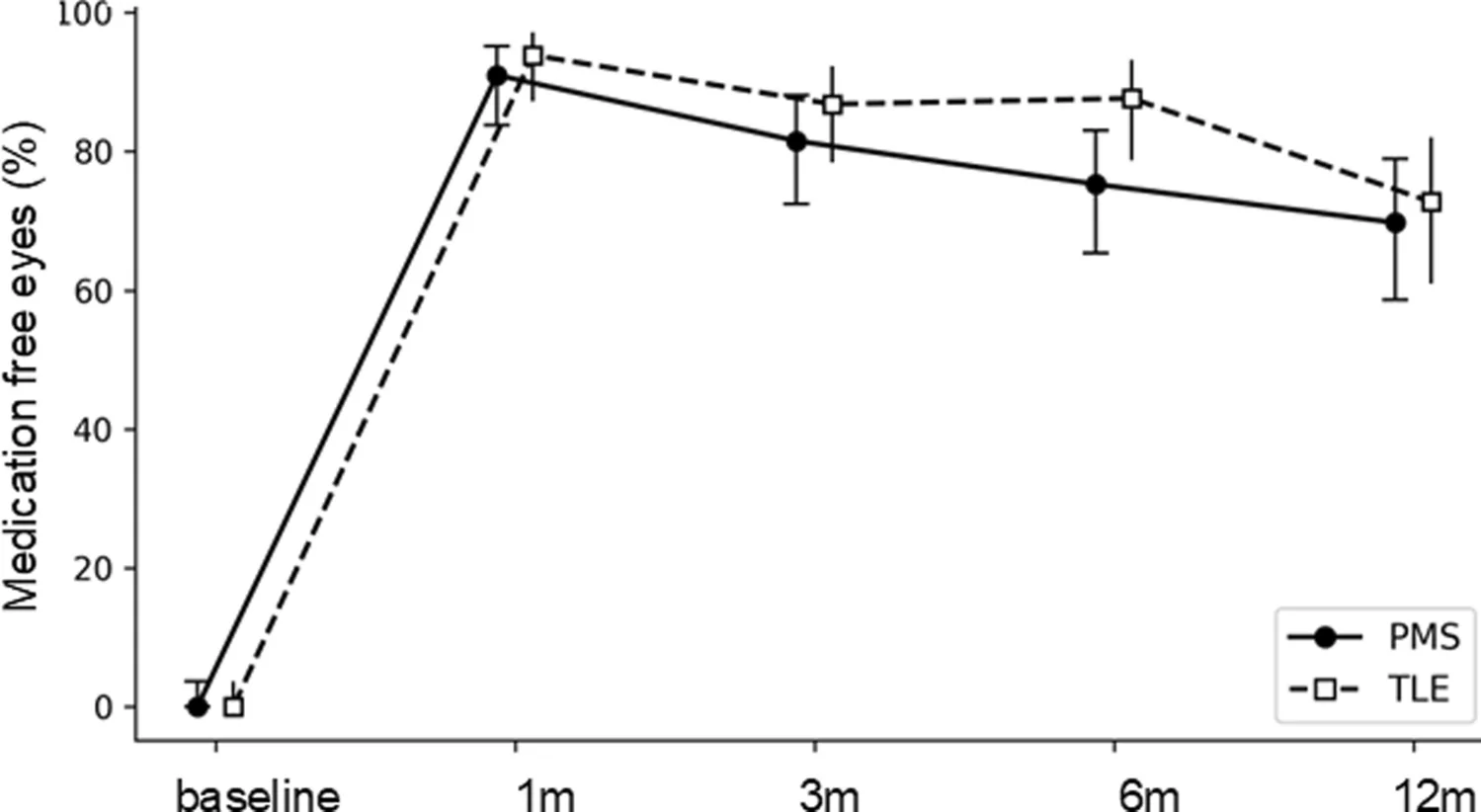
Postoperative time course of medication‑free status (PMS vs TLE). The proportion of medication‑free eyes is shown over time. Error bars represent 95% confidence intervals (Wilson method). PMS, PreserFlo MicroShunt; TLE, trabeculectomy.

**Topical antiglaucoma medications.** At baseline, patients used multiple medications in both groups (median PMS 4.0 [IQR 3.0–5.0]; TLE 5.0 [4.0–5.0]). Postoperatively, medication use decreased sharply, with over 90% of eyes medication-free at 1 month (PMS 91.0%, TLE 93.9%; median 0.0 in both groups). Although the medication-free proportion gradually declined thereafter, it remained the majority at 12 months (PMS 69.7%, TLE 72.7%; median 0.0 [0.0–1.0]). Detailed medication distributions are shown in ESM 2, and the time-course of medication-free rates with 95% confidence intervals (Wilson method) is presented in Fig. 3.

**Fig. 4:**
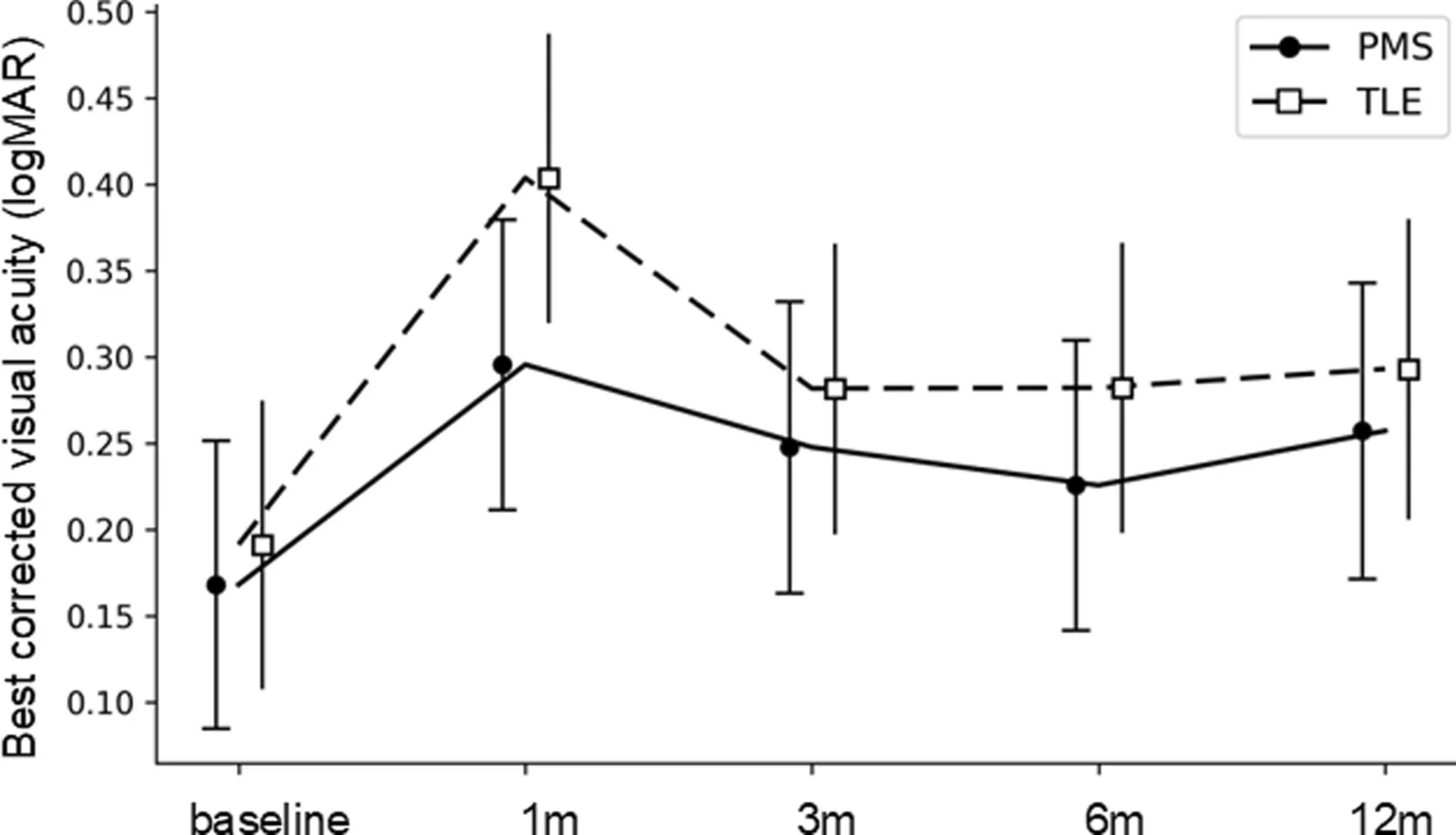
Estimated marginal mean best‑corrected visual acuity (logMAR) over time. Time‑course of best-corrected visual acuity (estimated marginal means with 95% CIs) at baseline and 1, 3, 6, and 12 postoperative months. PMS: solid line with filled circles; TLE: dashed line with open squares; horizontal offsets applied for clarity. Estimates are from a mixed‑effects model with a random intercept for matched pairs and AR(1) within‑subject covariance. PMS, PreserFlo MicroShunt; TLE, trabeculectomy; logMAR, logarithm of the minimum angle of resolution.

**Best-corrected visual acuity**. In the linear mixed-effects analysis (random intercept for matched pairs; AR (1) within-subject covariance), BCVA (logMAR) worsened at 1 month in both groups compared with baseline, followed by a partial recovery, but remained worse than baseline at 12 months. No significant between-group differences were detected at any postoperative time point (12 months: PMS 0.257 [95% CI, 0.171–0.343] vs TLE 0.293 [0.206–0.380]; difference PMS−TLE −0.036, 95% CI −0.151 to 0.080; Bonferroni-adjusted *p* = 0.541) (Fig. 4).

**Fig. 5:**
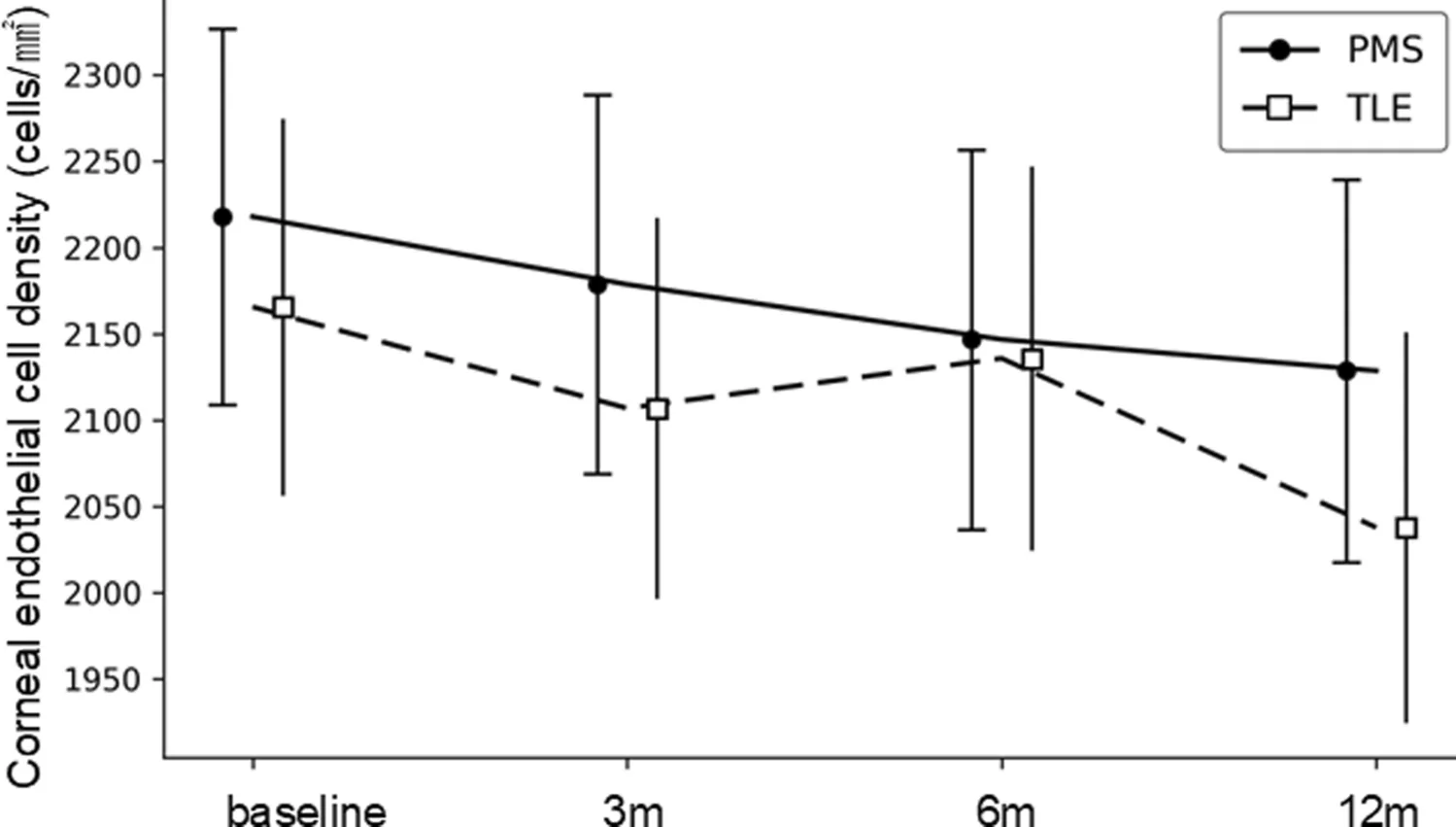
Corneal endothelial cell density over time. The plot shows estimated marginal means with 95% CIs at baseline, 3, 6, and 12 postoperative months; PMS: solid line with filled circles; TLE: dashed line with open squares. PMS, PreserFlo MicroShunt; TLE, trabeculectomy.

**Corneal endothelial cell density.** In the linear mixed‑effects analysis with a random intercept for matched pairs and AR (1) within‑subject covariance, ECD decreased over time in both groups (p < 0.001), whereas no significant main effect of procedure (PMS vs TLE) or procedure‑by‑time interaction was observed. Estimated marginal means showed a modest decline from baseline to 12 months in both PMS and TLE, with no significant between‑group differences at any time point (Fig. 5).

**Fig. 6:**
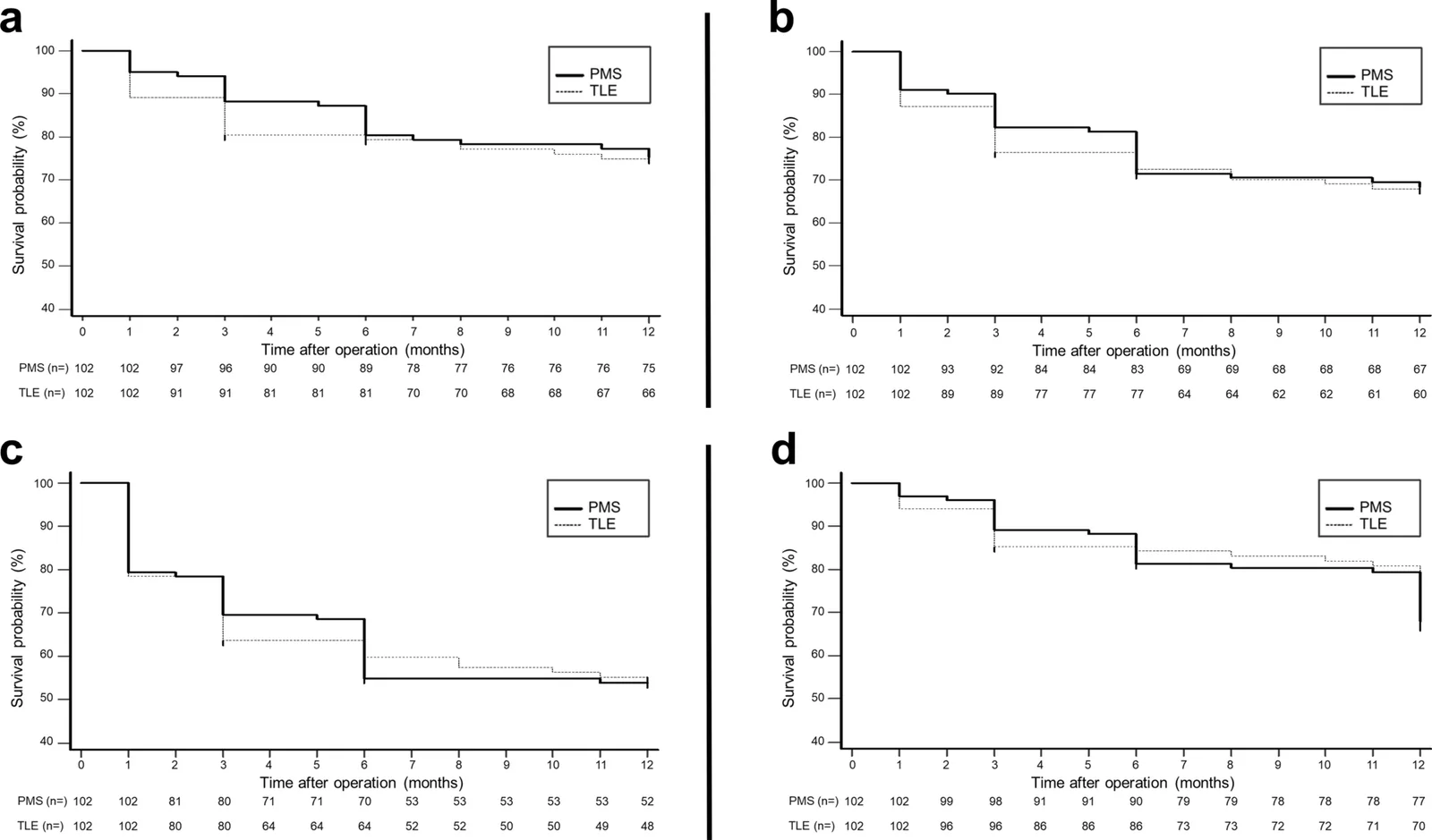
Kaplan–Meier curves for surgical success up to 12 months postoperatively. Surgical success was evaluated according to four different criteria: (a) criterion A, defined as an intraocular pressure (IOP) between 6 and 18 mmHg; (b) criterion B, defined as an IOP between 6 and 15 mmHg; (c) criterion C, defined as an IOP between 6 and 12 mmHg; and (d) randomized controlled trial (RCT)–based success criteria. Solid lines represent the PMS group, and dashed lines represent the TLE group. The number of eyes at risk (n) in each group is shown at the bottom of each graph. PMS, Preserflo MicroShunt; TLE, trabeculectomy.

**Success rates at 12 months postoperatively.** Success rates at 12 months were 75.2% (PMS) and 74.9% (TLE) for criteria (A) (Fig. 6a), 68.5% (PMS) and 68.0% (TLE) for criteria (B) (Fig. 6b), 53.9% and 55.1% for criteria (C) (Fig. 6c), and 69.1% (PMS) and 67.2% (TLE) for the RCT criteria (Fig. 6d). Overall, no significant differences in success rates were observed between the PMS and TLE groups for any criteria.

Table 3 shows the causes of surgical failure according to criterion C. Within 12 months, 47 eyes (46.1%) in the PMS group and 44 eyes (43.1%) in the TLE group met the criteria for failure. In both groups, the most common cause of surgical failure was insufficient IOP reduction. Additional surgical procedures leading to failure were performed in six eyes in each group. In the TLE group only, surgical failure occurred in six eyes due to prolonged hypotony and in two eyes due to complications requiring surgical intervention.

**Table 3:** Causes of failure based on criterion C

Causes of failure	PMS (n=102)	TLE (n=102)
Total (%)	47 (46.1%)	44 (43.1%)
Insufficient IOP reduction	41	30
Additional glaucoma surgery	6	6
Persistent hypotony	0	6
Complications requiring surgery	0	2

PMS, PreserFlo MicroShunt; TLE, trabeculectomy; IOP, intraocular pressure

Table 4 summarizes postoperative complications and additional surgery. Complications were significantly more frequent in the TLE group (64.7%) than in the PMS group (40.2%) (*P* < 0.001). The most frequent complication was hyphema, which occurred in 21 eyes in the PMS group and 30 eyes in the TLE group. Ten eyes in each group demonstrated a ≥20% loss of ECD, with no significant between-group difference. Hypotony-related complications occurred in 37.3% of eyes in the TLE group and 14.7% of eyes in the PMS group (P < 0.001). Hypotony-related events in the early postoperative period, including shallow anterior chamber, choroidal detachment, and hypotony maculopathy, were observed in both groups. However, prolonged hypotony persisting at 12 months postoperatively was observed only in the TLE group. Tube-related complications occurred in six PMS eyes (5.9%), including iris–tube adhesion, tube exposure, and transient tube occlusion by blood clot. Additional surgery was performed in 12 PMS eyes (11.8%) and 9 TLE eyes (8.8%), with no significant difference. In the PMS group, additional procedures included TLE (n=9), micropulse transscleral laser (n=1), and Ahmed valve implantation (n=2). In the TLE group, additional procedures included repeat TLE (n=2), Ahmed valve implantation (n=3), Baerveldt implantation (n=1), and surgery for complications (n=3), including amniotic membrane bleb revision, vitrectomy for malignant glaucoma, and vitrectomy for retinal detachment secondary to kissing choroidal detachment. Bleb revision was performed in 17 eyes in the PMS group and 22 eyes in the TLE group. Cataract surgery within 12 months after PMS or TLE surgery was performed in seven eyes in the TLE group, but in none of the eyes in the PMS group.

**Table 4:** Surgical complications and additional surgery

Surgical complications	PMS (n=102)	TLE (n=102)	*P* value*
Total (%)	41 (40.2%)	66 (64.7%)	**<0.001**
Hyphema	21	30	
Choroidal hemorrhage	0	1	
Bleb leakage (interventions)	1(0)	1(1)	
Macular edema (interventions)	1(0)	2(1)	
Malignant glaucoma	0	1	
Retinal detachment	0	1	
Reduction in ECD of over 20% from baseline	10	10	1
Complications due to hypotony	15 (14.7%)	38 (37.3%)	**<0.001**
Shallow anterior chamber (interventions)	4(3)	11(3)	0.10
Choroidal detachment	15	29	**0.03**
Hypotony maculopathy (interventions)	1(0)	4(1)	0.21
Prolonged hypotony (<6mmHg) at 12 months	0	5	**0.03**
Complications related to PMS tube	6 (5.9%)		
Pupillary deviation due to iris-tube adhesion	1		
tube exposure (interventions)	1 (1)		
Transient IOP elevation due to tube obstruction by blood clots	4		

Additional surgery			
Total (%)	12 (11.8%)	9 (8.8%)	0.65
TLE (PMS removal)	9 (6)	2	
Micropulse transscleral laser	1	0	
Ahmed glaucoma valve	2	3	
Baerveldt glaucoma implant	0	1	
Vitrectomy	0	2	
Bleb revision for leaking bleb with amniotic membrane	0	1	
**Bleb revision**	17	22	0.48

PMS, PreserFlo MicroShunt; TLE, trabeculectomy; ECD, corneal endothelial cell density; IOP, intraocular pressure. * Fisher's exact test

Bold values indicate statistically significant between‑group differences

## Discussion

This study is the first to compare one-year postoperative outcomes of PMS and TLE in Japanese patients with open angle glaucoma using propensity score matching to adjust for baseline differences. The primary outcome, IOP at 12 months, was significantly lower in the TLE group (12.3 mmHg) than in the PMS group (14.1 mmHg). These findings are consistent with the only existing RCT, in which Baker et al. report 12-month IOP values of 11.1 mmHg for TLE and 14.3 mmHg for PMS [9]. Although some studies report comparable IOP lowering effects between PMS and TLE [5, 6, 16, 17], the present results align with multiple previous reports, including RCTs and meta-analyses, demonstrating superior IOP reduction with TLE [4, 7–9, 11–13].

The outcomes of PMS in this Japanese cohort are consistent with previous domestic reports. The IOP in Japanese POAG eyes 6 to 12 months after PMS is reported to range from 11.3 to 14.1 mmHg [18–21]. Furthermore, in Japanese XFG eyes, it is reported to range from 14.8 to 16.9 mmHg [20, 22]. In our study, approximately 65% of eyes had POAG and 25% had secondary glaucoma, including XFG, and the PMS results fell within the range of prior Japanese findings.

Regarding longitudinal IOP trends, no early postoperative differences were observed between groups; however, TLE achieved greater IOP reduction from 6 months onward. Similar patterns are reported by overseas studies [4, 9, 13], suggesting that TLE may provide more sustained long term IOP control. Indeed, in the RCT, TLE maintained IOP approximately 3 mmHg lower than PMS from 3 months onward, and the 5-year extension study reported a 4.6 mmHg difference favoring TLE [12].

Previous studies report that PMS requires more postoperative glaucoma medications than TLE [4, 7–9]. In contrast, our study found no significant difference in the proportion of medication‑free eyes within 12 months postoperatively. Variability in target IOP, surgeon‑specific criteria for resuming medications, and the inclusion of secondary glaucoma cases may partly explain this discrepancy.

In both groups, BCVA worsened in the early postoperative period and then gradually showed a tendency toward recovery; however, BCVA did not return to baseline levels at 12 months postoperatively. Previous studies report no postoperative deterioration in visual acuity, as well as findings similar to ours, in which visual acuity temporarily worsened after surgery but subsequently recovered to baseline levels [11, 13, 16, 19]. In this respect, our results are inconsistent with most previous reports. Differences in patient characteristics and baseline visual acuity may have influenced these outcomes; however, further studies are required to clarify the factors associated with incomplete postoperative visual recovery.

ECD showed no significant overall reduction in either group, although 10 eyes in each group exhibited ≥20% loss. Previous studies report mixed findings regarding long term ECD changes after PMS or TLE. Pillunat et al. report that 26 eyes each undergoing PMS or TLE for POAG, ECD showed no difference from preoperative levels in either group at 6 months postoperatively [6]. Skowronski et al. report no reduction in ECD after 3 years of follow-up in 150 eyes undergoing PMS [23]. Conversely, a 5-year follow-up report of the RCT shows a reduction in ECD of approximately -19% compared to preoperative levels in both groups [12]. Similarly, a study of 8 Japanese POAG eyes undergoing PMS shows a significant reduction in ECD after approximately 5 years [18]. Importantly, no evidence currently suggests that PMS causes greater ECD loss than TLE.

In an overseas RCT [9], the surgical success rates at 12 months postoperatively were 53.9% in the PMS group and 72.7% in the TLE group, with more favorable outcomes observed in the TLE group. In the present study, however, when success was evaluated using criteria comparable to those of the RCT, the success rates were 69.1% in the PMS group and 67.2% in the TLE group, with no significant difference between groups. Because the RCT included only patients with POAG, we performed a subgroup analysis restricted to POAG patients in our cohort. In this analysis, the success rates were 76.4% in the PMS group and 70.3% in the TLE group, again showing no significant difference between the two groups. Furthermore, no significant differences in surgical success rates were observed between the PMS and TLE groups under any of the three criteria defined by upper IOP limits of 18, 15, and 12 mmHg.

A retrospective study by Fu et al., which included patients with secondary glaucoma and thus had patient characteristics similar to those of our study, reports success rates of 68.2% in the PMS group and 62.1% in the TLE group using a success criterion with an upper IOP limit of 21 mmHg, with no significant difference between groups; these findings were consistent with the results of the present study [4]. Given that many retrospective studies, including the present study, have reported comparable success rates between PMS and TLE, whereas a difference was observed in the RCT; the possibility that retrospective study designs remain influenced by residual confounding, even after the use of propensity score matching, cannot be excluded.

Notably, in the present study, as described above, bleb revision was treated in the same manner as needling and was not classified as an additional surgical procedure. This is because bleb revision at our institution is performed through a small incision with minimal invasiveness and is conceptually closer to needling than to conventional bleb revision procedures. When bleb revision was considered an additional surgery, the surgical success rates were 69.2% in the PMS group and 63.9% in the TLE group according to criterion A, 63.3% in the PMS group and 59.0% in the TLE group according to criterion B, and 50.1% in the PMS group and 52.8% in the TLE group according to criterion C (ESM 3).

Postoperative complications were significantly more frequent in the TLE group, particularly hypotony related events. Persistent hypotony and hypotony related complications contributed to surgical failure in several TLE eyes. These findings are consistent with previous reports. Transient postoperative hypotony after PMS is also reported, although less frequently than after TLE, with reported incidence rates ranging from approximately 10% to 50% [24]. In the present study, early postoperative hypotony‑related events, including shallow anterior chamber, choroidal detachment, and hypotony maculopathy, were also observed in the PMS group.

Although PMS is designed to provide controlled aqueous outflow, several previous studies describe hypotony and its sequelae following PMS implantation. Therefore, the relatively low incidence and transient nature of hypotony-related complications observed in the present PMS cohort should be interpreted with caution and may not be generalizable to all clinical settings. Differences in surgical technique, perioperative management, and patient background may partly explain the variability in reported hypotony rates across studies.

To prevent postoperative hypotony after PMS, several studies report the effectiveness of intraluminal stenting using nylon sutures or similar materials [24–28]. At our institution, intraluminal stenting with nylon sutures is selectively performed in eyes considered to be at high risk for postoperative hypotony. However, the cohort included in the present study did not contain any cases in which intraluminal stenting was used.

In contrast, persistent hypotony was observed only in the TLE group. Among the five eyes with persistent hypotony, defined as an intraocular pressure of <6 mmHg at 12 months postoperatively, two eyes had developed choroidal detachment in the early postoperative period, which had resolved by 12 months. One eye exhibited hypotony maculopathy that did not improve during follow‑up; however, no subjective decrease in visual acuity was reported. In the remaining three eyes, although IOP values met the numerical definition of hypotony, no clinically significant adverse findings were observed.

Although the rate of additional surgery did not differ between groups, all additional surgery in the PMS group was performed to further reduce IOP, whereas one third of additional surgery in the TLE group was for complications. Furthermore, cataract surgery within 12 months occurred only in the TLE group, potentially indicating a tendency toward a higher risk of cataract progression after TLE; however, the indication for postoperative cataract surgery is ultimately determined at the discretion of the operating surgeon.

This study has several limitations. First, this was a single-center retrospective study with a relatively short follow-up period of one year. Second, the study periods differed between the PMS and TLE groups. After the introduction of PMS at our institution, many cases that would previously have been indicated for TLE were instead treated with PMS, resulting in a reduction in the number of TLE procedures. Consequently, TLE cases performed during the same period as PMS included a higher proportion of secondary glaucoma, angle-closure glaucoma, and pediatric cases, leading to substantial differences in baseline patient characteristics compared with the PMS group. For this reason, the TLE group in the present study consisted of cases treated before the introduction of PMS.

In addition, during the initial introduction of PMS and the subsequent first year, learning curve–related effects and changes in postoperative management strategies, including the timing of postoperative interventions, may have influenced outcomes in the PMS group. Surgiical procedures and postoperative management were performed by multiple surgeons, which may have introduced inter-surgeon variability and potential bias, particularly for trabeculectomy, known to be highly technique-dependent. Although all surgeons involved had a minimum of three years of experience in glaucoma surgery, differences in individual experience and surgical technique could not be fully accounted for in this retrospective analysis.

Furthermore, secondary glaucoma cases were included, and diurnal IOP fluctuations, visual field mean deviation progression, and retinal nerve fiber layer thickness (RNFLT) changes were not analyzed. Cost-effectiveness, which has previously been reported to favor PMS [5], was also not evaluated. In addition, prior studies report conflicting findings regarding visual field and RNFLT progression after PMS versus TLE. Therefore, future prospective studies with longer follow-up and comprehensive structural and functional assessments are needed to provide a more complete comparison between these procedures.

In conclusion, PMS was associated with a more favorable safety profile, particularly with respect to hypotony‑related complications, whereas TLE achieved greater IOP reduction at 12 months. Surgical success rates were comparable between the two procedures. PMS may represent a less invasive option for Japanese patients with open‑angle glaucoma, while TLE may be more appropriate when maximal IOP lowering is a clinical priority. Longer-term studies incorporating structural and functional outcomes are warranted to further clarify the relative benefits of these two filtration surgeries.

## Supplementary Information

Below is the link to the electronic supplementary material.

Supplementary file1 (PDF 114 KB)

Supplementary file2 (PDF 112 KB)

Supplementary file3 (PDF 194 KB)
