# Supplementary material for: Comparison of one-year postoperative outcomes between PreserFlo MicroShunt and trabeculectomy in Japanese patients with open angle glaucoma: a retrospective cohort study using propensity score matching
Source: Jpn J Ophthalmol. 2026 May 30;70(5):1241–51. doi: 10.1007/s10384-026-01374-9 (PMC13615904; doi:10.1007/s10384-026-01374-9)
Supplement: Supplementary file 1 [file 10384_2026_1374_MOESM1_ESM.pdf]

Comparison of one-year postoperative outcomes between Preserflo MicroShunt and trabeculectomy in Japanese patients with open angle glaucoma: a retrospective cohort study using propensity score matching

ESM 1. Patients' demographic and ophthalmic data before propensity score matching

	**PMS (n=126)**	**TLE (n=174)**	***P* value**
Age (years)	74.0 (64.0 to 81.0)	70.0 (60.0 to 78.0)	**0.04**
Sex (male, %)	67 (53.2%)	100 (57.5%)	0.48*
Glaucoma type (POAG, %)	82 (65.1%)	102 (58.6%)	0.28*
pre IOP (mmHg)	21.0 (17.0 to 26.0)	24.0 (17.0 to 32.0)	**0.04**
pre MD (dB)	-16.3 (-21.9 to -11.0)	-16.8 (-21.7 to -11.8)	0.88
pre medication score	4.0 (3.0 to 5.0)	5.0 (4.0 to 6.0)	**0.001**
pre ECD(cells/mm2)	2376.0 (2012.0 to 2648.0)	2326.5 (1969.0 to 2639.0)	0.63
pre logMAR	0.000 (0.000 to 0.155)	0.097 (-0.079 to 0.398)	0.08

Data are shown as median (interquartile range).

PMS, Preserflo micro shunt; TLE, trabeculectomy; POAG, primary open angle glaucoma;
XFG, exfoliative glaucoma; IOP, intraocular pressure; MD, mean deviation;
ECD, endothelial cell density; log MAR, logarithm of the minimum angle of resolution.

Mann-Whitney test. * Fisher's exact test.

MD value was available in 72 eyes in PMS group and 111 eyes in TLE group.
