# Supplementary material for: Comparison of one-year postoperative outcomes between PreserFlo MicroShunt and trabeculectomy in Japanese patients with open angle glaucoma: a retrospective cohort study using propensity score matching
Source: Jpn J Ophthalmol. 2026 May 30;70(5):1241–51. doi: 10.1007/s10384-026-01374-9 (PMC13615904; doi:10.1007/s10384-026-01374-9)
Supplement: Supplementary file 2 [file 10384_2026_1374_MOESM2_ESM.pdf]

Comparison of one-year postoperative outcomes between Preserflo MicroShunt and trabeculectomy in Japanese patients with open angle glaucoma: a retrospective cohort study using propensity score matching

ESM 2. Time-course of the number of antiglaucoma medications (eyes, %) by surgical group

				Distribution (%) of patients by number of eye drops used			
**Surgery**	**Time**	**median[IQR]**	**eyes**	**0**	**1**	**2**	**3 or more**
**PMS**	Baseline	4.0 [3.0–5.0]	102	0 (0.0%)	1 (1.0%)	9 (8.8%)	92 (90.2%)
	Month1	0.0 [0.0–0.0]	100	91 (91.0%)	5 (5.0%)	2 (2.0%)	2 (2.0%)
	Month3	0.0 [0.0–0.0]	92	75 (81.5%)	7 (7.6%)	6 (6.5%)	4 (4.3%)
	Month6	0.0 [0.0–0.0]	89	67 (75.3%)	8 (9.0%)	2 (2.2%)	12 (13.5%)
	Month12	0.0 [0.0–1.0]	76	53 (69.7%)	6 (7.9%)	4 (5.3%)	13 (17.1%)
**TLE**	Baseline	5.0 [4.0–5.0]	102	0 (0.0%)	1 (1.0%)	8 (7.8%)	93 (91.2%)
	Month1	0.0 [0.0–0.0]	98	92 (93.9%)	0 (0.0%)	3 (3.1%)	3 (3.1%)
	Month3	0.0 [0.0–0.0]	91	79 (86.8%)	1 (1.1%)	4 (4.4%)	7 (7.7%)
	Month6	0.0 [0.0–0.0]	81	71 (87.7%)	2 (2.5%)	1 (1.2%)	7 (8.6%)
	Month12	0.0 [0.0–1.0]	66	48 (72.7%)	3 (4.5%)	5 (7.6%)	10 (15.2%)

Values are reported as eyes (%); IQR, interquartile range;

PMS, PreserFlo MicroShunt; TLE, trabeculectomy.
