# Supplementary material for: Comparison of one-year postoperative outcomes between PreserFlo MicroShunt and trabeculectomy in Japanese patients with open angle glaucoma: a retrospective cohort study using propensity score matching
Source: Jpn J Ophthalmol. 2026 May 30;70(5):1241–51. doi: 10.1007/s10384-026-01374-9 (PMC13615904; doi:10.1007/s10384-026-01374-9)

Comparison of one-year postoperative outcomes between Preserflo MicroShunt and trabeculectomy in Japanese patients with open angle glaucoma: a retrospective cohort study using propensity score matching

ESM 3. Kaplan–Meier curves for surgical success when bleb revision was considered an additional surgery

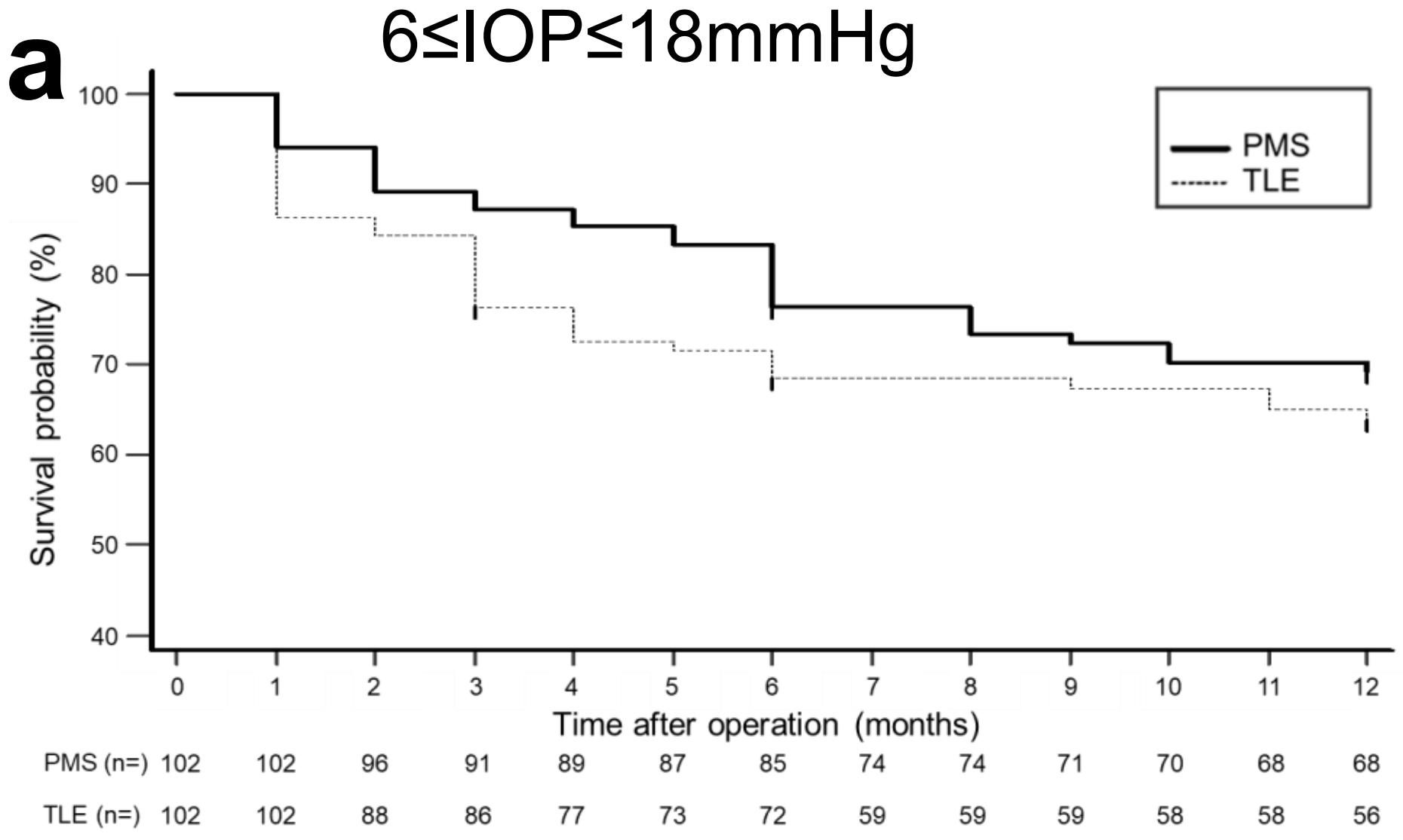

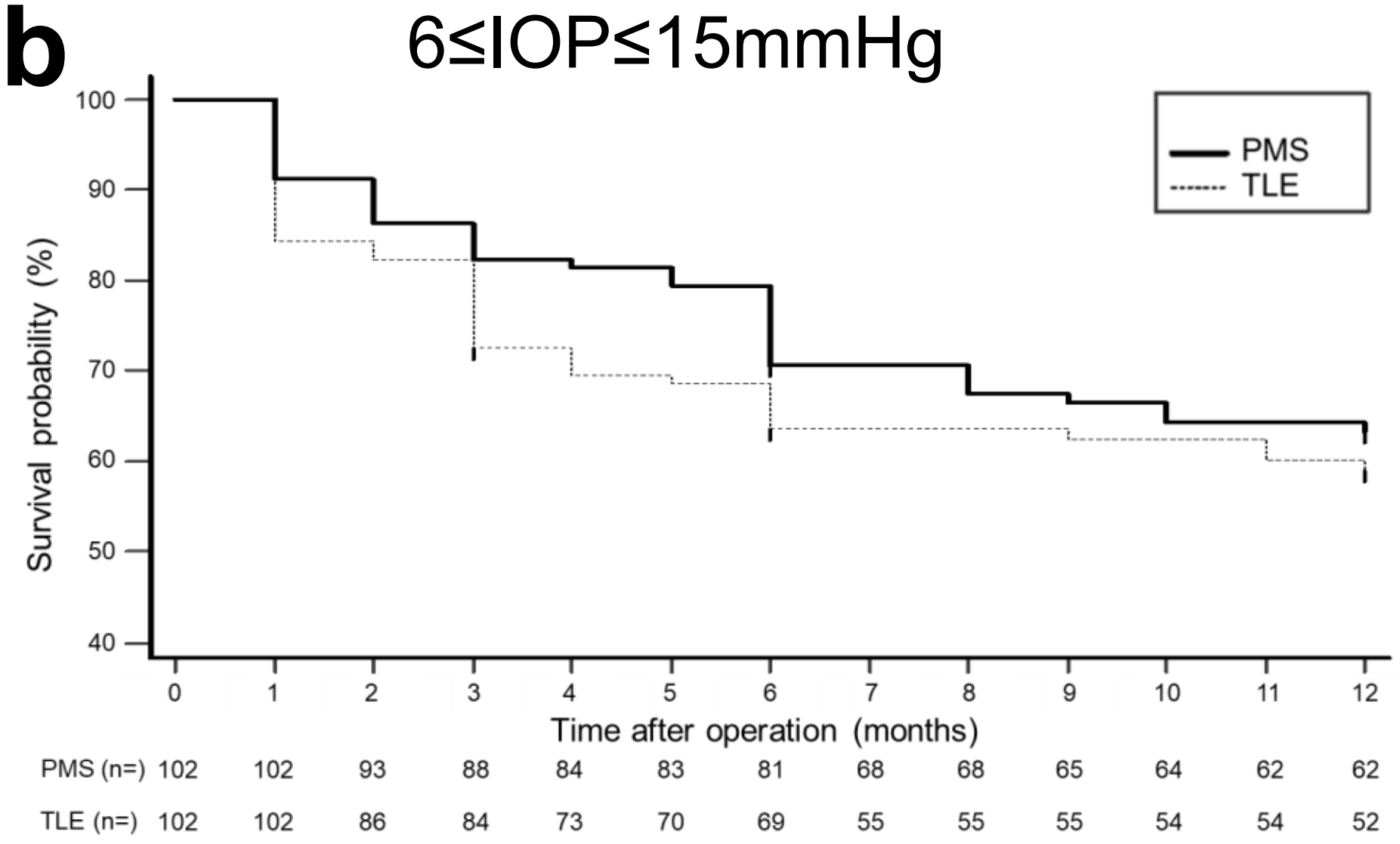

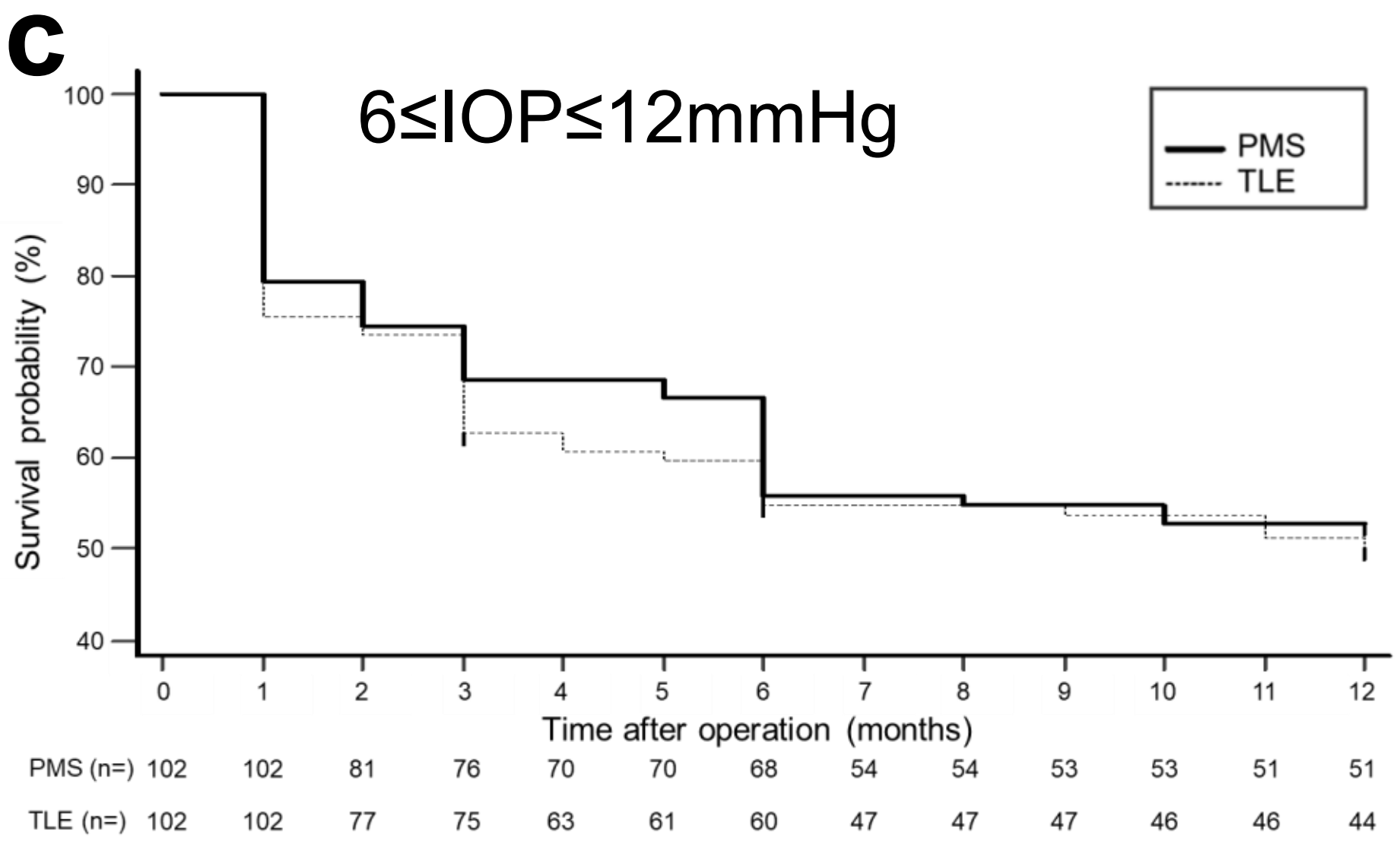

Supplement: Supplementary file 3 [file 10384_2026_1374_MOESM3_ESM.pdf]
